# Editorial: Mental practice: clinical and experimental research in imagery and action observation

**DOI:** 10.3389/fnhum.2015.00573

**Published:** 2015-10-15

**Authors:** Magdalena Ietswaart, Andrew J. Butler, Philip L. Jackson, Martin G. Edwards

**Affiliations:** ^1^Psychology, University of StirlingStirling, UK; ^2^Neuroscience Institute, Georgia State UniversityAtlanta, USA; ^3^École de Psychologie and CIRRIS and CRIUSMQ, Université LavalQuébec, Canada; ^4^Institut de Recherche en Sciences Psychologiques, Université Catholique de LouvainLouvain-la-Neuve, Belgium

**Keywords:** mental practice, action simulation, action observation, imagery, cognitive neuroscience

This editorial accompanies 18 articles as part of a *Frontiers* research topic. The aim of this research topic was to clarify the underlying mechanisms involved in mental practice of action, bringing together evidence from a range of disciplines including cognitive neuroscience, experimental neuropsychology, sport and movement science, clinical neuropsychology and clinical neurology. The need to clarify the underlying mechanisms of mental practice is a pressing one. Mental practice of action has been explored in sport psychology for several decades, with the aim to use mental practice to improve sport performance. However, following the discovery of the mirror neuron system (see for example, Rizzolatti and Craighero, 2004), the perspective of mental practice has changed to a rationale based on neuroscience and to research focussed on understanding the neural processes of mental practice. Evidence that the brain simulates action has resulted in a common understanding of “functional equivalence” (Jeannerod, 1994): the idea that the *mental* representation of an action or percept in the person's mind is the neural “equivalent” to the *physical* action or *actual* percept. This ability to mentally represent action using the motor system allows for action simulation, providing conscious mental rehearsal of movement (imagery), but also allows for a common percept when observing the movements of others. Finally, in recent years, the disciplines of clinical neuropsychology and neurology have begun to use mental rehearsal of action, or *mental practice*, to produce improvements normally attributed to practicing actual movements.

At the heart of all of the research is the idea that mental practice of action uses equivalent neural processes to those used in action execution. Of course, there is debate on what one understands to be “equivalent,” but the common reasoning seems to be that because mental practice (motor imagery and action observation) is functionally or neurally equivalent to actual practice, the efficacy principle of mental practice is that the motor areas are “trained,” perhaps through Hebbian learning “firing-rewiring.” Although the scientific foundation of this idea of action simulation is very sound in neuroimaging research (e.g., Sharma and Baron, 2013, this issue), the link to behavioral evidence or efficacy is currently weak. The neural correlates of mental practice are just that: correlates and do not justify inference about function, efficacy, or critical causality. There nevertheless seems to be reluctance in the field to address the underlying mechanisms of mental practice efficacy. This comes maybe as no surprise. A functional equivalence rationale for mental practice is intuitive and appealing and will therefore attract interest and funding. It is hardly in the researchers' interest to potentially undermine the idea by getting to the bottom of the matter.

We are now 15 or maybe 20 years into mental practice efficacy research based on the neural equivalence premise (Jackson et al., 2001). What is apparent is that the above simple interpretation of equivalence is not reflected in emerging data. It seems that mental practice efficacy is much more complex than simple Hebbian learning. There may be an analogy with the development of our understanding of the supplementary motor area (SMA) over that same time period. Initially SMA was thought of as a simple planning neural strip, but we have since understood the operation of SMA to be highly complex in the way it is involved in inhibition. For example, in studies using fMRI, motor imagery and action observation often do not activate the primary motor cortex (M1) because the SMA is thought to supresses the M1 activity (presumably preventing the individual from actually executing actions). The inherent role of inhibition in mental practice and the complexity of efficacy mechanisms still require further research. The popular notion that anything to do with the mirror neuron system is a simple matter of equivalence, or similarly that in applied contexts of mental practice equivalence is the end of the conversation, needs to change. We now need to go beyond what we are comfortable with and challenge what we know, even if we risk undermining the last decades of research.

There are still a lot of things that we do not know about the mechanisms of mental practice of action. What does each part of the brain engaged in mental practice actually do; how do processes relate to one another; what happens when different areas in the network are damaged? There are indications that insufficient drive to address the fundamentals of mental practice is starting to become a real issue of concern. The systematic review in this issue by Braun et al. (2013) concludes that the clinical evidence for mental practice efficacy in neuro-rehabilitation is weakening. The reasons for this seems to be the lack of theory-driven interventions, conceptual confusion (what does mental practice actually entail in practice?) and general methodological malaise including feasibility, dose, responders/non-responders, and adherence issues in larger scale trials that are more representative of clinical practice. Alternatively, when neuroscience evidence is carefully implemented in theory-driven clinical evaluation of mental practice, this may not translate to earlier reported clinical benefit (Ietswaart et al., 2011). Indeed, Malouin et al. (2013) in this issue highlight significant issues with the translation of experimental findings into clinical practice. Malouin et al.'s critical review is constructive, however, by suggesting ways in which the value of mental practice can be redeemed by addressing underlying mechanisms of mental practice efficacy. They conclude that the field must now truly put the use of mental practice to the test. Mental practice may indeed benefit the large number of stroke patients in neuro-rehabilitation, but unless mental practice is truly put to the test, this application may be superseded by other clinical innovations, for example, robotic assisted therapy. The field needs to deliver the necessary clarity on what exactly are the “active ingredients” of mental practice; what are the things that do not work and are mere distractions; which complexities play a role. Only then can we formulate effective guidance on what mental practice should actually entail in clinical practice. In the meantime, mental practice therapy in neuro-rehabilitation is already currently recommended treatment in many clinical guidelines. This current position means that we need to act fast in order to understand the processes and benefits of mental practice for clinical use. However, the current questionable guidance, range of possible uses, lack of efficacy etc. will likely undermine clinicians' willingness to adopt the treatment in the forthcoming years unless some clarity emerges.

Currently, much of the research effort goes to further documenting the correlates of mental practice, i.e., the fact that imagery and observation resonate with other motoric processes. In that respect, a number of the studies reported in this issue are exceptions to this rule in the way these studies ambitiously delineate the mental practice process by for example comparing the quasi-visual and the verbal-cognitive element of mental practice efficacy (Saimpont et al., 2013, this issue), or by contrasting the efficacy of different visual perspectives in mental practice (Callow et al., 2013, this issue; Yao et al., 2013, this issue), or by separating the impact of active imagery and passive observation (Eaves et al., 2014, this issue). It is an issue of concern, however, that such experimental approaches are generally not pursued (nor funded) as part of clinical evaluations, when now is the time to establish the finer details of mental practice efficacy in clinical contexts. We therefore advocate more high risk, high gain evaluations of mental practice that can establish the real impact of mental practice on the lives of real people in the clinic.

Further to bringing clarity with regards to the underlying mechanisms of mental practice, there is a real need to establish the modes of delivery and dosage. Clinicians furthermore need tools to make predictions of which patients will benefit and from what types of mental practice treatment. Lack of clarity on patient characteristics such as motor imagery ability can easily lead to miss-use of current findings exposing a risk of clinicians dismissing patients who they believe would not stand to benefit from mental practice–based rehabilitation. It would be great if we could say with some level of certainty whether a brain-damaged patient has an intact ability to use and benefit from mental practice therapies. Some authors would claim this can be done either through subjective methods such as vividness questionnaires, or through more objective methods such as mental chronometry (Milner, 1986), or monitoring automatic covert action simulation such as the cognitive hand mental rotation task established by Parsons (1987), or the response of the autonomic nervous system in mental practice as proposed by Collet et al. (2013, this issue). There is pressure on the research community to provide reliable measures of motor imagery ability on which clinicians can base a decision whether to provide a patient with mental practice rehabilitation. But quite possibly we do not (yet) have reliable tools on which such important decisions can be based. A study by de Vries et al. (2013, this issue) documenting motor imagery ability in stroke patients, showed that poor motor imagery ability as measured by subjective vividness questionnaires was not associated with poor performance also on *objective* imagery ability assessment. So although vividness scores suggested the patients had poor motor imagery, objective task performance in these stroke patients suggested that motor imagery was in fact intact. This situation could lead to the risk that clinicians when using only vividness scores could dismiss patients as poor imagers and therefore unable to benefit from mental practice-based rehabilitation, while the patients' imagery ability would be deemed intact if measured in other ways. Although Lawrence et al. (2013, this issue) report that high motor imagery vividness is associated with an increased benefit of mental practice in novice gymnasts compared to the lower performance gains in those with low motor imagery vividness, this relationship may not be a simple one suitable for rehabilitation treatment decisions.

This research topic aimed to address confusion regarding the concepts of imagery and observation which has hampered the progression of mental practice research both scientifically and in translation to clinical practice. Wondrusch and Schuster-Amft (2013, this issue) remarkably point to the need to address any confusion regarding mental practice even at a therapeutic level. They advocate a good understanding of theory and practice in recipients using mental practice rehabilitation techniques by describing ways to teach stroke patients mental practice. Other contributions in this issue broaden the concept of mental practice in a number of ways, such as Howatson et al.'s rationale for including the observation of one's own movements within the mental practice concept (Howatson et al., 2013, this issue), Smith and Wakefield's considerations with regards to the timing rate of mental practice (Smith and Wakefield, 2013, this issue), Kirsch et al.'s link between action simulation and aesthetic experience (Kirsch et al., 2013, this issue), Schack et al.'s novel theory of how mental practice develops cognitive mental representation structures (Schack et al., 2014, this issue), and importantly Vogt et al.'s meticulous review of the evidence of why mental practice should encompass both motor imagery and action observation (Vogt et al., 2013, this issue).

Because neuroimaging studies provide strong evidence for action simulation, but the link to behavioral change is perhaps weak, we invited contributions to show that mental practice efficacy might be driven by neuroplasticity processes evoked by action simulation. The preliminary work by Olsson and Lundstrom (2013, this issue) shows that successful action anticipation, as a precursor of mental practice, appeared associated with motor and temporal regions of the brain. Future research needs to investigate evidence of the associations between mental practice performance benefits and brain plasticity in the motor network. It is possible that combination of techniques is needed, including functional magnetic resonance imaging (fMRI), diffusion tensor imaging (DTI), MEG, and EEG.

In conclusion, in an attempt to build on interdisciplinary consensus on the nature and application of mental practice, this research topic integrated perspectives from the full range of the disciplines involved in mental practice research. It furthermore intentionally did not seek to limit mental practice to a narrow interpretation of conscious mental rehearsal of movement or motor imagery, but instead advocates to include imitation and action observation of self or others as an interpretation of mental practice as Action Simulation Therapy (AST). Such an interpretation of AST mental practice is justified in light of the evidence for neural equivalence. What the neuroscience of neural equivalence means for our understanding of behavior, mechanisms, and applied efficacy of mental practice, however, needs a much more sustained research effort devoid of complacency and supported by high-risk-high-gain research funding. With this shared and funded research drive it will be possible to accelerate our understanding and agreement on the core processes of mental practice, and therefore speed up the translation of evidence-based benefit of applied use of mental practice in sport and clinical practice.

## Conflict of interest statement

The authors declare that the research was conducted in the absence of any commercial or financial relationships that could be construed as a potential conflict of interest.
